# A qualitative analysis of statements on motivation of applicants for medical school

**DOI:** 10.1186/1472-6920-14-200

**Published:** 2014-09-23

**Authors:** Anouk Wouters, Anneke H Bakker, Inge J van Wijk, Gerda Croiset, Rashmi A Kusurkar

**Affiliations:** VUmc School of Medical Sciences, Institute for Education and Training, Post box 7057, 1007 MB Amsterdam, The Netherlands; LEARN! research institute for learning and education, Faculty of Psychology and Education, VU University, Amsterdam, The Netherlands; Department of Paediatrics, VU University Medical Center Amsterdam, Amsterdam, The Netherlands

**Keywords:** Admissions, Medical school selection, Motivation, Self-determination theory, Qualitative methods

## Abstract

**Background:**

Selection committees try to ascertain that motivated students are selected for medical school. Self-determination theory stresses that the type of motivation is more important than the quantity of motivation. Autonomous motivation, compared to controlled motivation, in students leads to better learning outcomes. Applicants can express their motivation in written statements, a selection tool which has been found to elicit heterogeneous responses, hampering the comparison of applicants. This study investigates the content of applicants’ statements on motivation for medical school in particular, the possibility to distinguish the type of motivation and the differences between selected and non-selected applicants.

**Methods:**

A thematic analysis was conducted on written statements on motivation (n = 96), collected as a part of the selection procedure for the graduate entry program for medicine and research at our institution. Themes were identified as motivation-related and motivation-unrelated (additional). The motivation-related themes were further classified as autonomous and controlled types of motivation. Group percentages for each theme were compared between selected and non-selected applicants using Chi-square test and Fisher exact test.

**Results:**

Applicants mainly described reasons belonging to autonomous type of motivation and fewer reasons belonging to controlled type of motivation. Additional themes in the statements included previous work experience and academic qualifications, ambitions, expectations and descriptions of the program and profession, personal qualities, and personal history. Applicants used strong words to support their stories. The selected and non-selected applicants did not differ in their types of motivation. Non-selected applicants provided more descriptions of personal history than selected applicants (p < 0.05).

**Conclusions:**

The statement on motivation does not appear to distinguish between applicants in selection for medical school. Both selected and non-selected applicants reported mainly autonomous motivation for applying, and included a lot of additional information, which was beyond the scope of what was asked from them. The findings raise a question mark on the validity and reliability of the statement on motivation as a tool for selection. It could however be of added value to enable applicants to tell their story, which they appreciate, and to create awareness of the program, resulting in an informed decision to apply.

## Background

One of the aims during selection procedures for medical school is to get an idea of the applicants’ motivation [1, 2]. Selection committees try to assess this through a description of applicants’ motivation in interviews, multiple mini interviews (MMI), personal statements, etc. [3, 4]. However, a reliable assessment of motivation could be difficult to realize in high stakes situations [5, 6], such as selection for medical school. A personal statement is a type of selection tool that often appears to evoke heterogeneous responses from applicants. In this study it is investigated whether statements on motivation as part of a selection procedure differ between selected and non-selected applicants.

**Figure 1:**
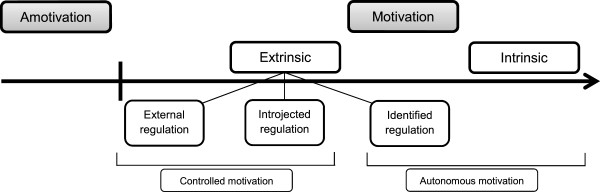
**The Self-determination continuum of motivation (adapted from** [8] **).**

By assessing the applicants’ motivation for medical school and the medical profession, selection committees want to ensure that the selected students are motivated. Self-determination theory (SDT; [7–9]) stresses that the type of motivation is more important than the quantity of motivation. It describes motivation as a continuum comprising of different states, namely a lack of motivation, motivation because of external factors and motivation because of internal factors (Figure 1). Autonomous motivation originates from within the individual and represents a sincere interest in (intrinsic motivation) and a positive personal valuation of (identified regulation) the study of medicine. Controlled motivation results from internal pressures, such as feelings of guilt or shame (introjected regulation), and external pressures, such as monetary rewards or parental pressure (external regulation). Autonomous motivation, in comparison with controlled motivation, in students has been found to result in better learning outcomes [10–15]. Moreover, the quality of motivation is a predictor of persistence and dropout [16].

In selection procedures in many medical schools, applicants are asked to express their motivation for the medical study in a personal statement. Some studies have found personal statements to yield heterogeneous responses, complicating the comparison of applicants [3]. Though it is widely used, research on the personal statement’s reliability and ability to predict future performance is inconclusive [17–24]. In line with this, a more specific writing assignment used in selection procedures, the essay question, has been found to evoke a similar heterogeneity of responses. Applicants provide responses that went beyond the scope of the posed question, in order to “show themselves” and “tell their own story”, a phenomenon which is expected to occur regardless of the official topic [25].

In some selection procedures a statement on motivation is included. In personal statements, applicants are expected to write about a variety of issues relevant to their application, whereas a statement on motivation has a particular focus on motivation. Analysis of statements on motivation has never been done before. The current study adds to the literature by investigating the content of applicants’ statements on motivation for medical school and the ability of these statements to distinguish between applicants. The research questions were: 1) what do applicants write in their statements on motivation for medical school?, and 2) do statements on motivation of selected applicants differ from those of non-selected applicants? Statements on motivation will be analysed from an SDT perspective to identify autonomous and controlled types of motivation, and to examine whether selected applicants describe their motivation differently from non-selected applicants. Additional occurring themes will also be identified.

## Methods

### Setting

**Figure 2:**
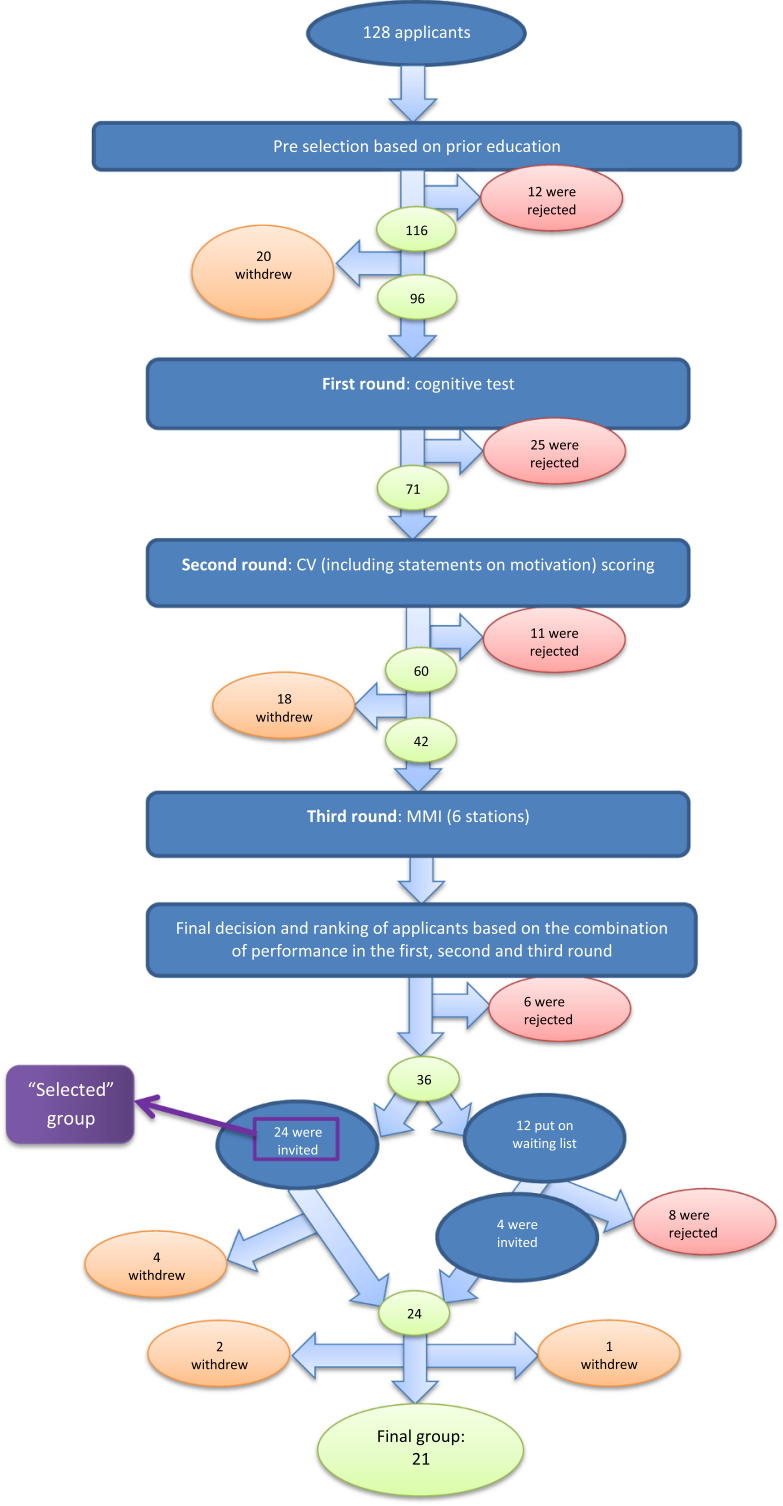
**Flow chart of the graduate entry selection procedure.**

This study was based on the analysis of applicants’ statements on motivation for admission into a graduate entry program in medicine and research at VUmc School of Medical Sciences, Amsterdam. Applicants for this program need to have a Bachelor’s degree in Biomedical Sciences or Health Sciences. The statement on motivation was a part of the selection procedure in 2012. Of the 128 initial applicants, 116 met the educational qualifications criteria and were invited to participate in a three step selection procedure, which consisted of a basic science cognitive test, scoring of application forms (containing prior academic achievement, like GPAs, and affinity and experience with scientific research and health care) and a 6-station Multiple Mini Interview (Figure 2). Ultimately, the 24 best scoring applicants, based on their performance at all steps of the selection procedure, were offered admission to the program. As a part of the application form, submitted prior to the cognitive test, applicants were asked the following: *“In max. 200 words, give an explanation of your motivation for applying for this graduate entry program at VUmc School of Medical Sciences”*.

### Data collection

Ninety-six statements on motivation were available for analysis. In order to capture all occurring themes, all submitted personal statements on motivation were included in this study after being anonymised. The statements on motivation were collected during the selection procedure, maximising the authenticity of the context for data collection. The analysis was carried out retrospectively, independent of the selection procedure.

### Data analysis

A constructivist paradigm was chosen, acknowledging the subjectivity of the researchers [26]. The researchers considered the context in which the data were constructed, namely a high stakes selection procedure, and were aware of the possible influence of their prior knowledge of applicants’ approaches towards selection tools based on the literature. One researcher (IW), as the coordinator of the graduate-entry program, was involved in the selection procedure and was therefore not involved in the data analysis. The other researchers were not involved in the selection procedure.

Data were analysed in a deductive way for descriptions of motivation and other themes described by applicants simultaneously (because it was expected, based on the literature, that the statements on motivation would also contain themes beyond the scope of the assignment). After that a categorisation of the descriptions of motivation into autonomous and controlled types was conducted. Occurrence of the types of motivation and additional themes in the statements was then compared to identify differences between selected and non-selected applicants.

For identifying all themes described by applicants, a thematic analysis [27] was conducted on the statements on motivation by two researchers (AW and AB). AW is a PhD student in medical education, conducting research on selection and motivation, and was familiar with the literature on personal statements during the analysis. AB is a policy advisor on postgraduate training and was only slightly familiar with personal statements. Both researchers have an educational background in psychology and were not involved in the selection procedure. Analyses were conducted independently to identify keywords and phrases representing categories of the topics that were described. This provided the basis for a coding scheme, which was discussed and adjusted during the analysis when necessary. The coding scheme, as well as the observed patterns and new occurring categories, were monitored through the use of memo-writing and discussed between the researchers during several meetings throughout the analysis. Whenever a new category was identified, all data were screened again for occurrence of that category. Categories were clustered to form overarching themes, which were finalized through discussion and consensus. For example, the categories *ambition for personal growth, ambition to contribute to society,* and *specified future work area* together formed the overarching theme *ambitions.* All the research team members were involved in the final findings discussion, so there were enough checks and balances in arriving at the results.

The data on motivation were further categorised into autonomous and controlled motivation using the framework of SDT by AW and RK (an expert on SDT). Within autonomous motivation, quotes were identified as intrinsic motivation or identified regulation. Within controlled motivation, quotes were identified as external regulation or introjected regulation.

Frequency analysis was conducted for the occurrence of all identified themes, and of the different types of motivation of selected and non-selected applicants. Group percentages were compared using a Chi-square test, or a two-sided Fisher's exact test when some expected frequencies were less than 5, in order to investigate the ability of the identified themes and motivation types to distinguish between selected applicants and non-selected applicants.

### Ethical approval

Ethical approval for this study was obtained from the Ethical Review Board of the Dutch Association for Medical Education (NVMO-ERB, file number 257).

## Results

Thematic analysis was conducted for all 96 statements. Eighteen of the 96 applicants withdrew their applications during the selection procedure, because they were admitted to a medical program in another school. Their statements were excluded from the analysis of frequency, as there was no way of knowing whether they would have been selected or not. The selected group consisted of the 24 applicants that were ultimately offered admission. The non-selected group consisted of 54 applicants who were rejected during the selection process or who were admitted from the waiting list, because these applicants would have been rejected if all 24 applicants who were initially invited to the program had accepted the offer of admission (see Figure 2).

Of the 96 applicants that wrote a statement on motivation, 27 were male (28.1%) and 69 were female (71.9%). The gender distribution of the selected group and the non-selected group was 5 males (20.8%), 19 females (79.2%) and 17 males (31.5%), 37 females (68.5%), respectively. The mean age was 23 (SD = 2) for 95 of the 96 applicants (as for one applicant information on age was not available), 22 (SD = 1) for the selected group and 23 (SD = 2) for the non-selected group.

The themes that occurred in the applicants’ statements on motivation are described and illustrated with quotes that were agreed upon by the research team as being representative for the findings. While analysing the data, two types of information were recognized. One was a description of participants’ motivation, as was intended by the selection committee. The other concerned additional personal information which seemed to be included to convince the selection committee to select the applicant. This covered a wide range of themes. There was overlap between themes, which will be addressed below.

### Motivation

Applicants described why they wanted to study medicine or become a doctor and/or researcher. These descriptions appeared to be in line with the published program description.

*Example: “The program of the VU University leads you to become not only a doctor, but also a researcher. Both professions appeal to me, but it is especially the combination of the two that I am interested in”.*

Applicants also provided statements of their enthusiasm and their strength of motivation, or they just expressed that they were motivated.

*Example: “I have a lot of passion, motivation and drive for medicine, but also for research”.*

They referred to their interests related to the study and profession, e.g. interest in the human body or helping people or doing research or the social aspect or specific specialties, etc. Applicants sometimes explained why these were their topics of their interest.

*Example: “Since childhood I have been interested in people and their (dys)functioning. What makes a person sick and how can I make it better keeps fascinating me”*

Applicants explained their choice for our institution, which could be driven by a preference for the organization of the institution or the attractions of the city.

*Example: “The VU University is well organized and has a well-structured curriculum. These were the deciding factors for me to choose the VU University”.*

Applicants’ motivation for applying could originate from personal life events.

*Example: “But the main reason why I want to become a doctor, is because I almost lost my mother when I was ten years old. […] I've seen what the medical community meant for my mother and me and then I knew I wanted to become a doctor”.*

The quotes in this theme were analysed further in order to identify autonomous motivation, i.e. doing something out of interest or enjoyment (intrinsic motivation) or because the behaviour is appreciated as being personally valuable (identified regulation) and controlled motivation, i.e. doing something for the promise of reward or the threat of punishment (external regulation) or because of experienced internal pressure, such as feelings of guilt and shame (introjected regulation).

Most (approximately 75%) of the reasons for applying for the graduate entry program described by the applicants could be categorised as autonomous motivation.

*Example: “The human body fascinates me, right from the molecular level to the body as a whole. The way our cells communicate mesmerizes me, and the more I read about the perfect physiological functioning of the human body, the more I want to know about it”.*

Within autonomous motivation, there were quotes that represented intrinsic motivation and identified regulation. Examples of intrinsic motivation are:

*“My intrinsic motivation is the interest and respect I have for the patient as a human being. My fascination with the human body plays a very big role in this”*

*“I compensate this [lack of experience in healthcare] with interest and motivation, and my affinity with science is greater than that of dopamine with its receptor”*

An example of identified regulation is

*“personal interaction with people is of great importance for me”.*

A reason that could be categorised as controlled motivation was

*“This program appeals to me because there might be more opportunities to start a PhD program during the Master education, and graduate earlier”.*

Within controlled motivation, there were quotes that represented external regulation.

*Example: “Next to the type of study, I would love to study in Amsterdam. I haven’t lived in a large (student) city in the Netherlands before, so I would love to experience living in the most famous Dutch city for a number of years”*

No quotes were found in the introjected regulation category.

The numbers of descriptions of autonomous and controlled motivation themes were compared between selected and non-selected candidates (Table 1). No significant differences were found.

**Table 1:** **Comparison of occurrence of descriptions of autonomous and controlled motivation between selected and non-selected applicants**

Type of motivation	Selected applicants	Non-selected applicants	Comparative statistics
Autonomous motivation	N = 49; 75.4%	N = 143; 74.9%	p = 0.934
Identified regulation	N = 32; 49.2%	N = 68; 35.6%	p = 0.052
Intrinsic motivation	N = 17; 26.2%	N = 75; 39.3%	p = 0.057
Controlled motivation	N = 11; 16.9%	N = 21; 11.0%	p = 0.212
Introjected regulation	N = 0; 0%	N = 0; 0%	
External regulation	N = 11; 16.9%	N = 21; 11.0%	p = 0.212

### Additional personal information

#### Previous work experience and academic qualifications

Applicants gave an overview of their prior education and work experience. They described how this had sparked their desire to become a doctor and researcher, what they missed and how the graduate entry program could fulfil their needs, and how these prior experiences made them suitable for the program.

*Example: “At the moment I am a third year Bachelor student Biomedical Sciences at the VU, in addition I have gained managerial experience, I work as a volunteer in healthcare and I am doing an internship at the child and adolescent psychiatry department of the VUmc”.*

#### Ambitions

Participants’ ambitions concerned personal growth or making a contribution to the society. Also, descriptions in this theme varied from a broad ambition of becoming a doctor or to help people, to a more specified intended work area for the future.

*Example: “By being scientifically engaged (especially with my background as a biomedical scientist), I want to make a contribution to medical science and development”.*

There seemed to be a distinction between applicants who described their desire to combine being a doctor and researcher, and applicants who mostly described their wish to become a doctor.

*Examples: “I would like to bridge the gap between scientific research and medical practice: “from bench to bedside”. Physician-scientist seems to be the designated profession for this”.*

*”My greatest wish is: ‘to become a doctor’!”*

#### Expectation and description of the graduate entry program and professionM

The program was addressed in terms of a description of the program characteristics, which often reflected the published program description. Applicants also described their expectations of the program. This concerned the expected challenging nature of the program, sometimes followed by a presentation of some of their personal qualities, to show how or why they would be able to handle this challenge. Descriptions also contained elaborations on the future profession, for example in terms of its societal relevance. Many applicants referred to how this program and profession would enable them to represent the link between science and health care, “theory and practice”.

*Examples: “The combination of physician and researcher in this program is a great addition to my education, to get the complete picture of illness, health and the human being as a whole. The approach to bring these domains together is crucial to provide optimal care to patients”.*

*“The graduate entry program of VUmc provides the possibility for conducting research full-time for a period of 8 months during the Master phase of medical education, and part-time during the clerkships”*

#### Personal qualities

Applicants listed their personal qualities, either with or without providing an explanation or describing situations in which they had shown these qualities. Promises for future performance were made, like how well they would behave or perform in their studies, or how well they would function as doctors. Some applicants displayed their need to portray their relevant knowledge, for example of the human body or chemistry. Statements of self-confidence were also observed in this theme, which could entail applicants expressing their suitability for the program and their expectation to be chosen.

*Examples: “If I get the chance to follow this program, I will make the maximum use of it. I think I'm fit as a candidate because I am passionate and critical, well capable of collaboration, and I can contribute to the learning process through active participation and discussions”.*

*“I would like to let you know that I am a suitable candidate for admitting into the graduate entry program of the VUmc, and for completing it with satisfactory results”*

*“Reliability, empathy, analytical ability, perseverance, and punctuality are the qualities that I pack in my suitcase for my medical journey”.*

#### Personal history

Personal details concerned life experiences, which could be related or unrelated to health care, the applicants’ application history, and “near and dear ones” working in health care. Life experiences related to health care were descriptions of how the applicant, a relative or a friend was struck by a disease and the health care they received, observed or witnessed. Life experiences unrelated to health care entailed, for example, stories on migration and difficulties experienced in previous education.

When describing their path towards the current application, applicants wrote about the strength of their desire to become a doctor. This was expressed either in terms of how early in their childhood they had identified this desire, or the effort they had invested to be able to study medicine (e.g. the purpose of engaging in certain educational activities), or the disappointment and consequences that followed former failed applications.

*Examples: “From early on in my high school years, I knew that I wanted to become a doctor. After finishing high school I enrolled into a pre-medical track … I knew that, after completing this, I would have a good chance of getting admission into a medical program. My secondary education and the University College have been a good preparation for the graduate entry program”.*

*“Ever since my childhood, I have had to deal with a lot of sickness in my immediate environment”*

*“As a political refugee from a country where people rarely have access to basic needs such as health care, and my experience with it, at a very young age I knew that I wanted to become a doctor and that I wanted to support less fortunate people”.*

*“To my great sorrow, I was not admitted to medical school the past 3 years”.*

#### Social aspect

Applicants described how the social nature of the medical profession appealed to them. This was often included in their description of what they missed in their current situation.

*Example: “…, the medical profession appeals to me, because I like to have personal contact with people”.*

#### Shortcomings in current situation

Applicants explained their current educational or professional situation and how this was not completely to their satisfaction. This often concerned their need for social interaction (with patients), which they expected to be fulfilled in the medical profession.

*Example: “I think research is fun, useful and important, but if I do only research, I miss the contact with patients and what do they experience. And especially the feeling that I can (directly) help someone, that I can cure someone, that’s what I miss”.*

#### Hope

Hope was expressed with regards to a successful outcome of the selection procedure. Applicants referred to their wish to proceed to the next step of the selection procedure or to be selected for medical school.

*Examples: “I hope you give me the chance to explain more in an interview”*

*“I really hope that I can participate in the next rounds of the selection procedure and I assure you of a 100 percent commitment to this study.”*

In addition to the above mentioned themes, the use of *strong words (superlatives)* was observed in almost all applicants’ writings.

*Examples: “The ultimate chance”*

*“An enormous drive of motivation”*

*”a lifelong exciting adventure in the medical world”*

*”I am 100 percent convinced that I was born to work with people”*

*“My dream, which gives an endless motivation”*

#### Overlap between themes

As can be seen in previous examples, some quotes contained more than one theme. Mainly, there was overlap between the *motivation* theme and one of the *additional personal information* themes, as applicants used the additional information to explain their motivation. This is illustrated by an example of the overlap between *motivation* and *personal history.*

*Example: “From the early years in my childhood, I have been confronted a lot of times with sickness in my immediate environment. This created a strong drive and passion in me to help people improve their health”.*

Within the *additional personal information* themes, especially the overlap between the *social aspect* theme and the *shortcomings in current situation* theme was common.

*Example: “I do follow my current Bachelor of Biomedical Sciences course with enthusiasm and great interest, but I miss the human interaction, the involvement with people and being able to mean something for them”.*

The occurrence of all themes that were identified in the personal statements of the selected and the non-selected applicants was calculated separately in order to establish whether the most successful applicants (from the whole selection procedure) mentioned different themes. Table 2 shows the number of statements on motivation in which the themes were mentioned for the selected and non-selected applicants, as well as percentages. P-values are reported for the differences between the groups. Non-selected applicants (33/54, 61.1%) more often described their personal history than selected applicants (8/24, 33.3%, p < 0.05). Differences for other themes were not statistically significant.

**Table 2:** **Occurrence of themes in the statements on motivation and comparisons between selected and non-selected applicants**

Theme	Selected applicants	Non-selected applicants	Comparative statistics
Motivation	N = 24	N = 50	p^§^ = 0.659^1^
	100%	92.6%	
Previous work experience and academic qualifications	N = 21	N = 48	p = 1.000^1^
	87.5%	88.9%	
Ambitions	N = 22	N = 45	p = 0.487^1^
	91.7%	83.3%	
Expectation and description of the graduate entry program and profession	N = 18	N = 43	p = 0.648^2^
	75.0%	79.6%	
Personal qualities	N = 20	N = 39	p = 0.291^2^
	83.3%	72.2%	
Personal history	N = 8	N = 33	p = 0.023^2^ *
	33.3%	61.1%	
Social aspect	N = 12	N = 23	p = 0.544^2^
	50.0%	42.6%	
Shortcomings in current situation	N = 8	N = 22	p = 0.535^2^
	33.3%	40.7%	
Hope	N = 5	N = 12	p = 0.891^2^
	20.8%	22.2%	
Strong words (superlatives)	N = 22	N = 52	p = 0.306^1^
	91.7%	96.3%	

^§^0.5 was added to each absolute value in order to enable Fisher exact test calculations.

^1^Calculations performed using Fisher exact test.

^2^Calculations performed using Chi-square test.

*Significant at p < 0.05 level.

## Discussion

This, to our knowledge, is the first study which reports about the type of motivation that applicants describe in statements on motivation used in a selection procedure for medical school. The reasons both selected and non-selected applicants provided for applying to the program were in line with findings from other studies [18, 21, 28–31] and concerned mainly autonomous motivation. There are two possible explanations for this finding. One is that those who apply to this program have higher autonomous motivation compared to controlled motivation. In another study (Wouters A, Croiset G, Galindo-Garre F, Kusurkar RA: Motivation of medical students: selection by motivation or motivation by selection, in preparation) on the selected population only (n = 21), we found high scores on autonomous motivation and moderate scores on controlled motivation (average scores of 6.19 and 4.12 on a Likert-scale of 1 to 7). Putting these results and those of the current study together, the large difference between reported autonomous and controlled types of motivation (75.4% autonomous motivation versus 16.9% controlled motivation), was not fully reflected in the motivation scores observed in the study on the selected population. This discrepancy indicates that applicants tend to emphasize their autonomous motivation and underreport their controlled motivation. This raises a question on the validity and reliability of a statement of motivation as a tool for selection. Similar behaviour was reported in research on essay questions [32]. A “hidden curriculum of admissions” was detected, described as ‘What do they want me to say’, which states that applicants estimate the expectations of the selection process and adjust their answers accordingly. The question remains whether a reliable assessment of motivation is possible in a high stakes situation such as selection.

Apart from the expressions of motivation, another type of information could be identified in the statements on motivation. This concerned information, which was beyond the scope of what was asked from them. Similar to findings from other studies, the inclusion of information about the personal qualities, prior education, and work experience of the applicants was observed [19, 29]. This type of information might have been included to show the applicant’s suitability for the medical program. We hypothesize that applicants provided a description of their personal circumstances, because they expected other applicants to be suitable as well, portraying similar qualities in their statements. The personal information might be perceived by the applicants as a means to convince the selection committee to select them over other applicants. Especially candidates who expect to fall short in terms of education and experience compared to the other candidates, might rely more on the impact their personal story has on the selection committee’s decision [29]. This could explain the difference in the number of statements of personal history between selected and non-selected applicants.

Without the ability to distinguish between applicants, could a written statement still be of added value in selection procedures? From the applicants’ point of view, the ability to tell their story and present themselves as unique individuals is much appreciated [25, 29]. Our results indicate that applicants read the website and/or flyers about the program, and used the information to write their statements. Thus, from the selection committee’s point of view, the statement can be a useful tool to make those who are interested in studying medicine aware of the program characteristics. Especially when students receive insufficient information on a medical career from their schools, this tool will encourage them to explore the course characteristics, in order to be able to make a well-informed decision on whether or not to apply [31].

A written statement is a “difficult to score” tool, because of the heterogeneity of responses. In addition, written statements are subject to embellishment [33]. Applicants are likely to provide socially desirable answers in such a high stakes situation [34, 35], and to get help in writing their statements [3, 18, 36]. Applicants turn to their peers, family or even professional agencies for input for their statements. Though written statements do not have a prominent role in selection in the Netherlands (which may reduce the likelihood of applicants turning to professional agencies), the findings on the content of the statements in this study are consistent with findings observed in countries where the personal statement is given more importance.

A strength of the present study was that some research team members were experts on SDT and others were not. This facilitated in-depth analysis of the data as well as identification of the whole range of themes unrelated to SDT, but important in getting an insight into the use of “statements on motivation” by applicants. This study has some limitations. First, the background of the researchers could have biased the analysis and interpretation of the data. In qualitative research however, and especially in accordance with the constructivist paradigm, reflection on the background of the researchers can benefit the research. Prior knowledge does not necessarily infer, and can even contribute to a more meaningful interpretation of the data. Second, although for the selected applicants we were able to compare findings from the current study with measurements on autonomous and controlled motivation from a previous study, such comparisons were not possible for the non-selected applicants. It is expected that selected and non-selected applicants show similar behaviour in response to a writing assignment during a selection procedure. A third limitation is the difficulty we experienced in categorising a few of the motivation quotes (especially the intrinsic motivation and identified regulation quotes). We resolved differences of opinion through discussion and consensus.

## Conclusions

This study provided an insight in the statement on motivation as part of a selection procedure for medical school. Both selected and non-selected applicants described mainly autonomous motivation for applying and were less elaborate on their controlled motivation. Our results suggest that a statement of motivation is not a valid and reliable tool for the assessment of motivation. The statement on motivation could be of value to make applicants aware of the program characteristics, resulting in an informed decision to apply, and to provide space for telling their story.

## Authors’ information

AW, MSc, is a PhD student in medical education at VUmc School of Medical Sciences.

AB, MSc, is the Head of Faculty Development and a policy advisor on postgraduate medical education at VUmc School of Medical Sciences.

IW, PhD, is coordinator of the graduate entry program in medicine and research at VUmc School of Medical Sciences.

GC, MD, PhD, is Professor in medical education and the Director of VUmc School of Medical Sciences.

RK, MD, PhD, is an Assistant Professor and the Head of Research in Education at VUmc School of Medical Sciences.

## Abbreviations

SDT: Self-Determination Theory
VUmc: VU University Medical Center
VU: VU University.

## References

[1] Turner R, Nicholson S (2011). Reasons selectors give for accepting and rejecting medical applicants before interview. Med Educ.

[2] Breland H, Maxey J, Gernand R, Cumming T, Trapani C (2002). Trends in College Admission 2000. A Report of a Survey of Undergraduate Admissions Policies, Practices, and Procedures.

[3] Albanese MA, Snow MH, Skochelak SE, Huggett KN, Farrell PM (2003). Assessing Personal Qualities in Medical School Admissions. Acad Med.

[4] Guyaux J, oude Egbrink MGA, Heeneman S, Houben AJHM, Willekes C, Schuwirth LWT, de Goeij AFPM (2010). Selectie op een combinatie van cognitieve en noncognitieve eigenschappen. Keuzes en ervaringen in de onderzoeksmaster Arts-Klinisch Onderzoeker (A-KO) te Maastricht. Tijdschrift voor Medisch Onderwijs.

[5] O'Neill LD, Korsholm L, Wallstedt B, Eika B, Hartvigsen J (2009). Generalisability of a composite student selection programme. Med Educ.

[6] Kusurkar R, Croiset G, Kruitwagen C, Ten Cate TJ (2011). Validity evidence for the measurement of the strength of motivation for medical school. Adv in Health Sci Educ.

[7] Deci EL, Ryan R (1985). Intrinsic motivation and self-determination in human behavior.

[8] Ryan RM, Deci EL (2000). Intrinsic and Extrinsic Motivations: Classic Definitions and New Directions. Contemp Educ Psychol.

[9] Ryan RM, Deci EL (2000). Self-determination theory and the facilitation of intrinsic motivation, social development, and well-being. Am Psychol.

[10] Sobral DT (2004). What kind of motivation drives medical students' learning quests?. Med Educ.

[11] Kusurkar R, Croiset G, Galindo-Garre F, Ten Cate TJ (2013). Motivational profiles of medical students: Association with study effort, academic performance and exhaustion. BMC Med Educ.

[12] Vansteenkiste M, Zhou M, Lens W, Soenens B (2005). Experiences of autonomy and control among Chinese learners: Vitalizing or immobilizing?. J Educ Psychol.

[13] Artino AR, La Rochelle JS, Durning SJ (2010). Second-year medical students's motivational beliefs, emotions, and achievement. Med Educ.

[14] Kusurkar RA, Ten Cate TJ, Vos CMP, Westers P, Croiset G (2013). How motivation affects academic performance: a structural equation modelling analysis. Adv Health Sci Educ.

[15] Kusurkar RA, Ten Cate TJ (2013). AM Last Page: Education is not filling a bucket, but lighting a fire: Self-Determination Theory and motivation in medical students. Acad Med.

[16] Vallerand RJ, Fortier MS, Guay F (1997). Self-determination and persistence in a real-life setting: toward a motivational model of high school dropout. J Pers Soc Psychol.

[17] Benbassat J, Baumal R (2007). Uncertainties in the selection of applicants for medical school. Adv Health Sci Educ.

[18] Hanson MD, Dore KL, Reiter HI, Eva KW (2007). Medical school admissions: revisiting the veracity and independence of completion of an autobiographical screening tool. Acad Med.

[19] Ferguson E, Sanders A, O'Hehir F, James D (2000). Predictive validity of personal statements and the role of the five-factor model of personality in relation to medical training. J Occup Organ Psychol.

[20] Ferguson E, James D, O'Hehir F, Sanders A, McManus IC (2003). Pilot study of the roles of personality, references, and personal statements in relation to performance over the five years of a medical degree. Commentary: How to derive causes from correlations in educational studies. BMJ.

[21] Dong T, Kay A, Artino AR, Gilliland WR, Waechter DM, Cruess D, DeZee KJ, Durning SJ (2013). Application essays and future performance in medical school: Are they related?. Teaching and learning in medicine.

[22] Oosterveld P, Ten Cate TJ (2004). Generalizability of a study sample assessment procedure for entrance selection for medical school. Med Teach.

[23] Dore KL, Hanson M, Reiter HI, Blanchard M, Deeth K, Eva KW (2006). Medical school admissions: enhancing the reliability and validity of an autobiographical screening tool. Acad Med.

[24] Peskun C, Detsky A, Shandling M (2007). Effectiveness of medical school admissions criteria in predicting residency ranking four years later. Med Educ.

[25] White JS, Lemay JF, Brownell K, Lockyer J (2011). "A Chance To Show Yourself" - how do applicants approach medical school admission essays?. Med Teach.

[26] Ng S, Lingard L, Kennedy TJ, Swanwick T (2013). Qualitative research in medical education: Methodologies and methods. Understanding Medical Education: Evidence, Theory and Practice.

[27] Braun V, Clarke V (2006). Using thematic analysis in psychology. Qual Res Psychol.

[28] Rolfe IE, Ringland C, Pearson S (2004). Graduate entry to medical school? Testing some assumptions. Med Educ.

[29] Ding H (2007). Genre analysis of personal statements: Analysis of moves in application essays to medical and dental schools. Engl Specif Purp.

[30] McManus IC, Livingston G, Katona C (2006). The attractions of medicine: the generic motivations of medical school applicants in relation to demography, personality and achievement. BMC Med Educ.

[31] Harrison R, Turney B, Blundell A (2003). Motivation and insight of students considering a career in medicine. Med Teach.

[32] White J, Brownell K, Lemay JF, Lockyer J (2012). What Do They Want Me To Say? The hidden curriculum at work in the medical school selection process: a qualitative study. BMC Medical Education.

[33] Kumwenda B, Dowell J, Husbands A (2013). Is embellishing UCAS personal statements accepted practice in applications to medicine and dentistry?. Med Teach.

[34] Mueller-Hanson R, Heggestad ED, Thornton GC (2003). Faking and selection: Considering the use of personality from select-in and select-out perspectives. J Appl Psychol.

[35] Griffin B, Wilson IG (2012). Faking good: self-enhancement in medical school applicants. Med Educ.

[36] Laurence CO, Zajac IT, Lorimer M, Turnbull DA, Sumner KE (2013). The impact of preparatory activities on medical school selection outcomes: a cross-sectional survey of applicants to the university of Adelaide medical school in 2007. BMC Medical Education.

[CR37] The pre-publication history for this paper can be accessed here:http://www.biomedcentral.com/1472-6920/14/200/prepub

